# Virtual psychiatric care fast-tracked: reflections inspired by the COVID-19 pandemic

**DOI:** 10.1192/bjb.2020.97

**Published:** 2020-08-17

**Authors:** Tea Rosic, Sandra Lubert, Zainab Samaan

**Affiliations:** 1 McMaster University, Hamilton, Ontario, Canada; 2 Hamilton, Ontario, Canada

**Keywords:** Telemedicine, telepsychiatry, virtual care, COVID-19, pandemic

## Abstract

For many patients and healthcare providers, the move to virtual psychiatric care has been fast-tracked by the COVID-19 pandemic. In this article, we consider a patient perspective and a provider perspective on the transition to virtual psychiatric care and its strengths and limitations, as well as a call for much-needed future research.

The COVID-19 pandemic has rapidly transformed service delivery, including the delivery of medical care, around the world. As jurisdictions worldwide enacted measures to slow the spread of the SARS-CoV-2 virus, service providers quickly adapted. What was previously a slow expansion of telemedicine and virtual care over the past two decades, with the primary goal of overcoming geographical barriers,^1^ became suddenly fast-tracked in a matter of weeks. A rapid transition to virtual psychiatric care, care provided by telephone or video, is one such transformation resulting from the COVID-19 pandemic and the measures implemented to contain it.

Leaders and advocates in the field of telepsychiatry are expecting, and in fact encouraging, these changes to persist post-pandemic.^2–4^ The aim of this article is to reflect on the transition to virtual psychiatric care through three lenses: a patient perspective, a provider perspective and a scholarly perspective, including a call for future research. The patient and provider perspectives obtained for this article reflect individual experiences with the transition to virtual psychiatric care and are not intended to capture the diversity of perspectives and experiences that may exist. The patient and providers have experienced both models of care pre- and post-pandemic onset.

## A patient perspective, by Ms Sandra Lubert

‘I am a mental health patient with a diagnosis of depression, PTSD and chronic sleep disruption. I have been either ill or in remission for many years and have experienced extensive clinical intervention both before and during the COVID-19 pandemic; that is, both actual, in-person care and virtual, online care.

Clearly, a physical meeting between patient and practitioner in a clinical environment is often warranted. Face-to-actual-face is sometimes best, and I am not advocating the replacement of such visits with online care. Further, some of our most vulnerable populations lack access to telehealth and/or virtual technology and for these individuals, it is imperative that we either provide them with the tools required to access proposed alternative care or continue to provide traditional, in-person interventions. Barriers to online care can be significant, salient features and these need to be considered. When a physical appointment is required or even simply preferred, this must be accommodated. I believe that an ideal system of mental health care features a robust, multi-faceted approach which meets the needs of all patients and practitioners.

My own mental illness includes severe insomnia and related suicidality. Six weeks ago – right around the onset of the pandemic – I had a psychiatric crisis requiring an ER visit and overnight hospitalization. This urgent, on-site care was life-saving. But I believe that my follow-up care, which has been entirely virtual, has been just as critical. This recent experience leads me to believe that the rapid changes necessitated by COVID-19 have a great deal to teach us about psychiatric care delivery.

For me, as a recipient, ‘pandemic-period psychiatry’ (via eVisits) has not only been adequate and helpful, it has in many ways been far more effective than its in-person counterpart. There are three main aspects of online care that have enhanced the overall experience for me: reduced cost, ease of access, and increased flexibility/comfort. From a very practical standpoint, I can state that eVisits have saved me money. I have not had to pay for gas to get to and from appointments, and I have not had to pay for parking. Many patients travel a significant distance in order to see clinicians, and this can be very expensive. Some rely on caregivers, taxis, or public transit. Again, while this may seem insignificant, the cost of transportation can be a major deterrent in seeking treatment, particularly for those on a limited income.

Ease of access around virtual care is a huge factor. My mental illness is often incapacitating; when I am in crisis, it is difficult for me to get out of bed, let alone leave my house. Things like getting dressed, planning for travel time, being presentable may seem trivial, but for those struggling with severe mental illness, these obstacles can be insurmountable. The anxiety induced by having to leave the house and get to one's doctor is sometimes crippling and prevents us from seeking care. When I am in distress, the idea of sitting in a waiting room (often in tears, usually in psychic pain) is intolerable. For those dealing with a physical disability in addition to mental illness, barriers are even more debilitating. When access is restricted, the situation with mental health can become dire. Indeed, it can even become deadly. eVisits have saved me; I have been better able to access consistent, necessary psychiatric care because I can do so virtually from my home.

Increased flexibility is perhaps the most beneficial aspect of virtual care. Since the variables of transportation and location are removed, both patient and practitioner are better able to schedule meetings. Modifying appointments has also been far easier to accomplish online, with recipient and caregiver both having immediate access to electronic calendars and other tools. Involving my partner in an eVisit is merely a matter of inviting her to sit next to me at my computer. Both my doctor and I have information at our fingertips if needed.

Finally, I feel more comfortable with eVisits. Meeting with my practitioner from my own environment puts me more at ease. Ultimately, this has to do with a levelling effect – it makes me feel like I am more of an equal in the patient–practitioner exchange. Traditional appointments take place in the doctor's realm – their office, clinic, the hospital… in some ways, this gives them the ‘home field advantage’, as it were. I am in their space. They know where everything is, who everyone is, but it is largely unfamiliar to me. I am a guest. I don't have my own mug, for tea.’

## A provider perspective, by Dr Tea Rosic, psychiatry resident, and Dr Zainab Samaan, staff psychiatrist

‘Suddenly, going to the clinic to see patients is a health risk to both my patients and me. I am faced with a precarious balancing act as I find ways to provide adequate psychiatric care to patients who face increasing anxiety, depression, social isolation, and changes in mental status during the pandemic. The novelty of COVID-19, the ever-dynamic guidelines, processes, and instructions, issues of shortage and conservation of PPE, and new risk–benefit equilibria as we consider each clinical decision are all present in ways we have not encountered before.

Virtual care has alleviated the risk carried by face-to-face contact but has raised many new challenges. Do I have a printer, fax machine, dedicated telephone line, secure email, and contact information for community pharmacies? Do I know how to schedule appointments without administrative staff support? New medicolegal challenges arise: what if a patient has an urgent psychiatric need, should I be available online 24 h/day? Can we send patients to hospital or are we contributing to risk of transmission and placing patients at greater risk? Not unlike before the pandemic, my patients in greatest need of care often face the greatest barriers in accessing it: limited mobile phone minutes, barriers in access to internet and virtual technologies, lack of privacy.

Nevertheless, virtual care has been transformative. Being able to provide much needed care despite the pandemic restrictions is satisfying. Seeing patients through telemedicine modalities has opened the door to better assessments of their environment and allows for easier involvement of other family members when invited. I see patients’ pets and other important aspects of their lives. How often does it occur, in the clinic, that we have no access to updated medication lists, and how much time is spent trying to gain this information before making further treatment recommendations? With virtual visits and medications accessible to the patient in their home, this problem is averted.

Virtual care during this pandemic has so quickly transformed how (and from where) we do our jobs. I have greater control over my schedule, working hours are more flexible and I am more available outside of structured clinical time. How these changes will evolve following the pandemic is unknown, but there is much to be learned and gained from this experience.’

## A scholarly perspective: the evidence base

Virtual psychiatric care has an established evidence base and has shown effectiveness in a variety of areas, including within different patient populations and in different clinical settings.^5^ Previous research suggests that building therapeutic rapport is just as effective virtually as it is in person.^5^ For clinicians thrust into providing virtual psychiatric care, the strengths and limitations of this model of service delivery are becoming clearer. Recognising and reflecting on these is just as important now as it will be post-pandemic, when organisations and providers decide on the models of care they will offer. In the post-pandemic era, in-person healthcare may indeed become ‘option B’ for many patients.^6^ We are working through the technical issues, concerns about confidentiality and provider payment obstacles that each slowed the advance of virtual care in the past. For many patients, as described above, virtual care provides the easier access, flexibility and comfort that is lacking from in-person, hospital- or clinic-based care. Some patients may be greatly benefited by ongoing virtual appointments.

For all of the potential benefits of virtual psychiatric care, there are shortcomings that must be considered. Physical examination cannot be conducted as usual and there may be challenges in comprehensively assessing physical appearance and functioning in virtual psychiatric assessments. Virtual care may impose additional barriers to assessment for patients presenting with certain symptoms, such as paranoid ideation,^7,8^ although a recent study on the use of telepsychiatry for first-episode psychosis suggests that 50% of patients reported telepsychiatry as a favourable modality for follow-up.^9,10^ Patients experience differential access to virtual platforms and technology, based on socioeconomic and other factors.^11^ In particular, patients with severe and persistent mental illness may face even greater barriers to accessing care virtually than the general patient population.^8^ Individuals living in close quarters with multiple family members may have insufficient space to talk while maintaining privacy and confidentiality.^11,12^ Older adults and individuals with disabilities may face challenges with access to technology, visual impairment or hearing impairment, creating barriers and gaps in care. Cultural factors in virtual care must be carefully considered and addressed. Patients who require language interpreters may be disadvantaged by virtual psychiatric care.^13^ With the expansion of virtual psychiatric care, we must be particularly mindful of the risk of widening the gap in access to care for patients who are marginalised or otherwise vulnerable. The broader issue of global healthcare access inequities in telepsychiatry is being raised.^14^

## Research and practice implications

In this article, we aimed to provide both patient and provider perspectives on the rapid and monumental shift to virtual psychiatric care that occurred, seemingly overnight, in many jurisdictions. However, the perspectives shared may be limited in their generalisability for settings outside of a well-funded public healthcare system or in places with limited existing capacity for the provision of telemedicine. We stress that the experiences of unique patients and unique providers, working in diverse clinical settings, managing different clinical presentations and operating in distinct healthcare systems worldwide might be critically different. The global healthcare community will benefit from hearing and learning from diverse experiences and perspectives.

As we rapidly usher in this new era of virtual psychiatric care, concerted efforts must be made to study and learn from our experiences. Research must be undertaken to examine the impact of these changes in psychiatric service delivery for different patient groups and different providers. Ensuring identification of individuals and groups whose needs are not met will be critical. Qualitative research that can capture the depth and detail of our human experiences with virtual psychiatric care will be necessary. Economic analyses of the costs and savings of this model will also be integral. There is some pre-pandemic evidence to suggest great potential cost savings with widespread use of telemedicine – including savings accrued from shorter time spent travelling and waiting, for both patients and providers.^15,16^

Clinical and research groups worldwide have begun to publish and share their experiences in implementing telepsychiatry during the COVID-19 pandemic for patient populations in various settings, including child and adolescent psychiatry,^17^ general out-patient psychiatry^11,13^ and in-patient psychiatry.^18^ Surveys of psychiatrists using telepsychiatry during the pandemic indicate benefits such as convenience and flexibility, as well as challenges in relation to the use of technology, impact on confidence in diagnosis and impact on therapeutic alliance.^13^ Authors are giving consideration to the experience of trainees and educators using telepsychiatry.^19^

The COVID-19 pandemic propelled us into a new era of virtual psychiatric care, and opened the door to a re-evaluation of how, and why, we provide mental healthcare in the ways we do. This door will remain open, post-pandemic, allowing us to rigorously evaluate, shape and refine our models of care to meet the needs of our patients as best as possible.

## About the authors

**Tea Rosic**, MD, is a Resident Physician in the Department of Psychiatry and Behavioural Neurosciences and a PhD student in the Department of Health, Evidence and Impact at McMaster University, Hamilton, Ontario, Canada. **Sandra Lubert**, BA, BEd, is a writer/author and educational consultant in Hamilton, Ontario, Canada. **Zainab Samaan**, MBChB, MSc, DMMD, MRCPsych, PhD, is Associate Professor of Psychiatry and Behavioural Neurosciences in the Department of Health Research Methods, Evidence, and Impact at McMaster University, and Director of the Clinician Investigator Program within the Mood Disorders Program, St Joseph's Healthcare, Hamilton, Ontario, Canada.
